# Dysregulated gene expressions of *MEX3D*, *FOS* and *BCL2* in human induced-neuronal (iN) cells from NF1 patients: a pilot study

**DOI:** 10.1038/s41598-017-14440-7

**Published:** 2017-10-24

**Authors:** Noriaki Sagata, Takahiro A. Kato, Shin-ichi Kano, Masahiro Ohgidani, Norihiro Shimokawa, Mina Sato-Kasai, Kohei Hayakawa, Nobuki Kuwano, Ashley M. Wilson, Koko Ishizuka, Shiori Kato, Takeshi Nakahara, Makiko Nakahara-Kido, Daiki Setoyama, Yasunari Sakai, Shouichi Ohga, Masutaka Furue, Akira Sawa, Shigenobu Kanba

**Affiliations:** 1 Department of Neuropsychiatry, Graduate School of Medical Sciences, Kyushu University, Maidashi 3-1-1, Higashi-ku, Fukuoka, 812-8582 Japan; 2 Departments of Psychiatry, Mental Health, Neuroscience, and Biomedical Engineering, Johns Hopkins University School of Medicine and Bloomberg School of Public Health, 600 North Wolfe St., Baltimore, MD 21287 USA; 3 Department of Dermatology, Graduate School of Medical Sciences, Kyushu University, Maidashi 3-1-1, Higashi-ku, Fukuoka, 812-8582 Japan; 4 Department of Clinical Chemistry and Laboratory Medicine, Graduate School of Medical Sciences, Kyushu University, Maidashi 3-1-1, Higashi-ku, Fukuoka, 812-8582 Japan; 5 Department of Pediatrics, Graduate School of Medical Sciences, Kyushu University, Maidashi 3-1-1, Higashi-ku, Fukuoka, 812-8582 Japan

## Abstract

Direct conversion technique to produce induced-neuronal (iN) cells from human fibroblasts within 2 weeks is expected to discover unknown neuronal phenotypes of neuropsychiatric disorders. Here, we present unique gene expression profiles in iN cells from patients with neurofibromatosis type 1 (NF1), a single-gene multifaceted disorder with comparatively high co-occurrence of autism spectrum disorder (ASD). Microarray-based transcriptomic analysis on iN cells from male healthy controls and male NF1 patients (NF1-iN cells) revealed that 149 genes expressions were significantly different (110 upregulated and 39 downregulated). We validated that mRNA of *MEX3D* (mex-3 RNA binding family member D) was lower in NF1-iN cells by real-time PCR with 12 sex-mixed samples. In NF1-iN cells on day 14, higher expression of *FOS* mRNA was observed with lower expression of *MEX3D* mRNA. Interestingly, *BCL2* mRNA was higher in NF1-iN cells on day 5 (early-period) but not on day 14. Our data suggest that aberrant molecular signals due to *NF1* mutations may disturb gene expressions, a subset of which defines continuum of the neuronal phenotypes of NF1 with ASD. Further translational studies using induced pluripotent stem (iPS) cell-derived neuronal cells are needed to validate our preliminary findings especially confirming meanings of analysis using early-period iN cells.

## Introduction

Neurofibromatosis type 1 (NF1: also known as von Recklinghausen disease) is a multifaceted disease, which shows a variety of physical symptoms including multiple café-au-lait spots, Lisch nodules, neurofibromas, scoliosis, and vision disorder^1–3^. NF1 patients also show a variety of mental symptoms such as mental retardation, epilepsy, and cognitive impairment / learning disorder^4,5^. Around half of NF1 patients show impaired social information processing and disturbed social behaviors^6–8^. In addition, twenty to thirty percent of NF1 patients are known to have autism spectrum disorder (ASD)^9–11^. These clinical reports have suggested some neurodevelopmental pathophysiology in the brains of NF1 patients.

Neurofibromin 1, coded by the *NF1* gene, is responsible for the pathophysiology of NF1. A recent rodent study using mouse neural stem cells (NSCs) has shown that dysfunction of neurofibromin 1 increases the protein level of BCL2 (B cell leukemia/lymphoma 2), an anti-apoptotic protein^12^. A strong link between Ras-GTPase and neurofibromin 1 has long been established^13^. However, some studies have also shown that neurofibromin 1 regulates not only Ras-GTPase but also adenylyl cyclases (ACs) in various cell types^14^. However, the detailed molecular basis of neurofibromin 1 functions via ACs has not been elucidated. Interestingly, a recent study using a zebrafish model of NF1 has shown that AC signaling pathway is associated with learning^15^.

To our knowledge, no study has shown whether such dysfunctions exist in human living neuronal cells of NF1 patients, due to the difficulty of directly analyzing human brain cells, including neuronal cells. Conversion techniques from somatic cells (non-brain-derived cells) into neuronal cells have been highlighted as useful translational research tools especially for brain disorders including psychiatric disorders^16,17^. Directly converted neuronal cells, called “induced-neuronal (iN) cells”, were originally developed from mouse fibroblasts transfected with three transcriptional factors: *Brn2*, *Ascl1*, and *Myt1l* (BAM factors)^18^. We have successfully produced iN cells from human fibroblasts using human BAM factors^19–21^. In addition, we have developed an optimized protocol for producing iN cells from adult patients in the present study.

The main purpose of the present pilot study is to clarify dysregulated gene expressions using comprehensive microarray-based transcriptomic analysis of iN cells from NF1 patients, especially via ACs in the presence or absence of forskolin, a typical ACs activator.

## Methods

All methods of this study were performed in accordance with the Declaration of Helsinki and were approved by the ethics committees of Kyushu University (Fukuoka, Japan). This report does not include any information that identifies information on individuals of healthy controls and patients.

### Materials

We established primary human fibroblasts from healthy volunteers and patients at Kyushu University Hospital, and also purchased human fibroblasts from the cellular bank of the Coriell Institute (Camden, NJ, USA) (Supplementary Table 1). Informed consent was obtained from all the healthy volunteers and patients before donating skin fibroblasts. To establish the fibroblast cell culture, the epidermis and subcutaneous adipose tissues were removed from biopsied skin tissue, and the remaining dermis tissue was placed on a clean culture dish. The dermis tissues were radially impressed the surgical knife to stick them to the bottom of the culture dish. Fibroblasts were maintained in Fibroblast Growth Medium (FGM) that contained 15% fetal bovine serum (FBS) (Japan Bioserum, Hiroshima, Japan), 0.1 mM MEM Non-Essential Amino Acids (NEAA) (Thermo Fisher Scientific, Waltham, MA, USA), and 1% Pen Strep (Thermo Fisher Scientific) in Minimal Essential Medium Eagle (MEM) (Sigma Chemical Co., St. Louis, MO, USA).

### Generation of induced-neuronal (iN) cells from human fibroblasts

The method of generating iN cells has previously been reported^19^. Briefly, on Day 0, lentiviruses were applied to fibroblasts to express each of the BAM factors (*BRN2*, *ASCL1*, *MYT1L*, MOI = 10 each) in FGM which contained 8 ug/mL of polybrene (Sigma Chemical) for 24 hours. On the next day (Day 1), the medium was changed with fresh FGM to remove the lentiviruses. Two days after transfection, the medium was changed every 3 days with iN Medium (10 ng/mL FGF2 (Peprotech, Rocky Hill, NJ, USA), 1 mM valproic acid (Sigma Chemical), 10 μM forskolin (optional) (Nacalai Tesque, Kyoto, Japan), 0.8% N2 supplement (Thermo Fisher Scientific), 0.4% B27 supplement (Thermo Fisher Scientific), 1% Pen Strep, 10 μg/mL blasticidin (Thermo Fisher Scientific) in Dulbecco’s Modified Eagle’s Medium (DMEM)/Nutrient Mixture F12 Ham (Sigma Chemical): Neurobasal medium (Thermo Fisher Scientific) = 4:1).

### Immunocytochemistry

The cells were plated on Matrigel (CORNING, Corning, NY, USA) coated NUNC Thermanox plastic coverslip (Thermo Fisher Scientific) in a 24-well plate. The cells were fixed with 4% PFA (Nacalai Tesque) for 15 min. at room temperature (R.T.). After washing thrice with PBS, the cells were incubated with 0.1% Triton-X100/PBS for 15 min. at R.T., rinsed with PBS thrice again, and treated with Blocking One (Nacalai Tesque) for 1 hr. at R.T. The blocking buffer was replaced with primary antibody solution (mouse anti-beta III tubulin antibody: T8660 (Sigma Chemical) 1/500 diluted with Can Get Signal immunostain Solution A (TOYOBO, Osaksa, Japan)), and the specimens were incubated for O/N (overnight) at 4 deg C. After washing thrice with PBS, secondary antibody solution (Goat anti-Rabbit igG (H + L) Cross-Absorbed Secondary Antibody, Alexa Fluor 568: A-11031 (Thermo Fisher Scientific) 1/500 diluted with Can Get Signal immunostain Solution A (TOYOBO)) was applied for 1 hr. at R.T. in the dark. The cells were washed with PBS thrice, and incubated with 1/10000 DAPI solution/PBS (DOJINDO, Kumamoto, Japan) for 10 min. at R.T. in the dark. After washing thrice with PBS, the coverslip was mounted onto a slide glass with antifade mounting reagents (Thermo Fisher Scientific).

### Quantitative real-time PCR

Real-time PCR was performed with LightCycler 480 real-time PCR system (Roche Diagnostics, Mannheim, Germany). The total RNA was extracted from cultured cells by a High Pure RNA Isolation Kit (Roche Diagnostics) according to the manufacturer’s protocol. cDNA was synthesized using a Transcriptor First Strand cDNA Synthesis Kit (Roche Diagnostics). Primers and probes were shown in Supplementary Table 2. Human GAPD or mouse ACTB of Universal Probe Library (Roche Diagnostics) was used as house-keeping control genes.

### Gene expression microarrays

The cRNA was amplified and labeled using Low Input Quick Amp Labeling (Agilent Technologies, Santa Clara, CA, USA) and hybridized to a 60 K Agilent 60-mer oligo-microarray using Sureprint G3 Human Gene Expression Microarray 8 × 60 K v2 (Agilent Technologies), according to the manufacturer’s instructions. All hybridized microarray slides were scanned by an Agilent scanner. Relative hybridization intensities and background hybridization values were calculated using Agilent Feature Extraction Software (9.5.1.1).

The raw signal intensities of all samples were log2-transformed and normalized by quantile algorithm with ‘preprocessCore’ library package^22^ on Bioconductor software^23^. We selected the probes, excluding the control probes, where the detection p-values of all samples were less than 0.01 and use them to identify differentially expressed genes. Finally we applied Linear Models for Microarray Analysis (limma) package^24^ of Bioconductor software, and obtained 149 genes. The criteria was that limma adj. p-value < 0.05 and absolute log-fold-change (|logFC|) > 2 between HC and NF1-iN samples. We identified 1 gene between HC and NF1 fibroblast samples on the same criteria.

### Nf1/Mex3d knockdown cells

Mouse neuroblastoma cell line Neuro2A cells were cultured in growth medium (10% FBS, 1% GlutaMAX (Thermo Fisher Scientific), 1% Pen Strep in DMEM (4.5 g/l Glucose: Nacalai Tesque)) in 12-well plates until the monolayer cell density reaches to the optimal ~50% confluency. The Medium was replaced with fresh differentiation medium (0.5% FBS, 1% GlutaMAX, 1% Pen Strep, 10 μM retinoic acid (Tocris Bioschience, Avonmouth, Bristol, UK), and 10 μM forskolin (optional) in DMEM (4.5 g/l Glucose)) 60 minutes before transfection. For transfection, the cells were incubated with 30 nM of Neurofibromin 1 or Mex3d siRNA (m) (sc-36037 or sc-149398: Santa Cruz Biotechnology, Santa Cruz, CA, USA) or 30 nM of Control siRNA-A (sc-37007: Santa Cruz) in GenMute siRNA Transfection Reagent for Neuro2A (SL100568-N2A: SignaGen Laboratories, Rockville, MD, USA). At 48 hours, cells were harvested and used for quantitative real-time PCR.

### Statistics

To determine the differences between the groups, t-test, one-way ANOVA followed by Tukey’s correction, or two-way ANOVA followed by Tukey’s correction or Sidak’s correction was used.

## Results

### iN cells derived from NF1 patients

**Figure 1:**
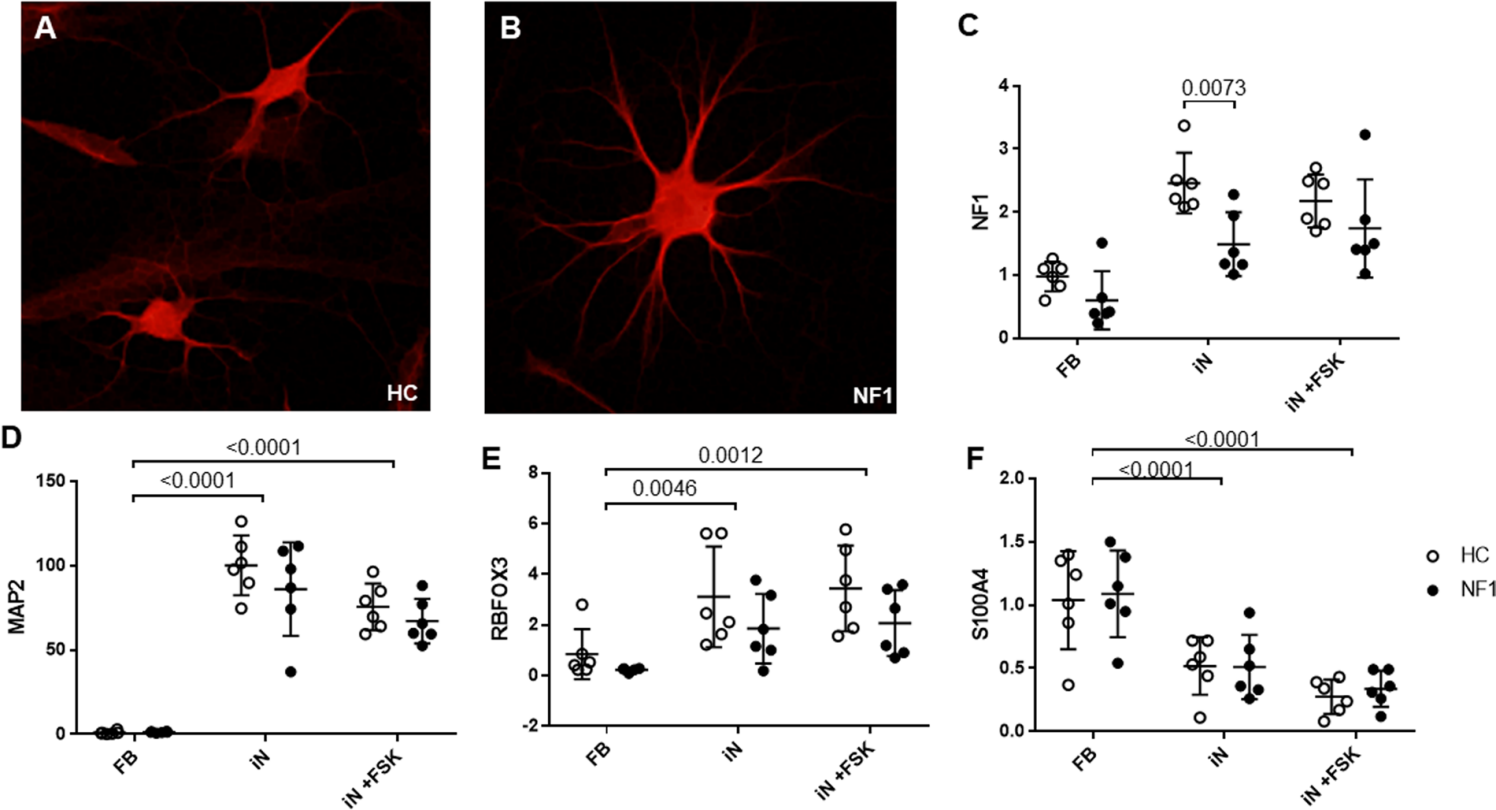
Expression levels of neuronal markers in iN cells at day 14 after the transfection of human BAM factors. Beta III tubulin (neuronal marker) positive iN cells derived from healthy control (**A**) and NF1 patient (**B**). (**C**) *NF1* mRNA expression levels were quantified by real-time PCR. Two-way ANOVA/Sidak’s test, n = 6 each group. (**D–F**) *MAP2* (pan-neuronal marker), *RBFOX3* (mature neuronal marker), and *S100A4* (fibroblast marker) mRNA expression level, respectively. Two-way ANOVA/Tukey’s test, n = 6 each group. Open circles show healthy controls and filled circles show NF1 patients.

We successfully produced iN cells from 6 healthy volunteers as Healthy Control (HC group) and 6 NF1 patients (NF1 group). Two weeks after the transfection of BAM factors to fibroblasts, we confirmed the existence of beta III tubulin, neuronal marker positive cells among a mixture of various cell morphologies (Fig. 1A,B). Conversion rate to neuronal cells using immunocytochemistry of MAP2 (Microtubule Associated Protein 2, a pan-neuronal marker) and DAPI (a nuclear marker) was about 10% (MAP2 positive/ DAPI positive cell). Total RNA was extracted from mixed culture dishes including these neuronal cells, and gene expression was quantified by real-time PCR (LightCycler 480 real-time PCR system: Roche Diagnostics, Mannheim, Germany). *NF1* mRNA expression level was significantly decreased by 39.4% in iN cells from NF1 patients (NF1-iN cells: Fig. 1C, p = 0.0073). Compared to fibroblasts culture (before the BAM factors transfection), *MAP2* and *RBFOX3* (RNA Binding Protein, Fox-1 Homolog 3: a mature neuronal marker) were significantly increased in 2 week-cultured cells after the BAM transfection (we define the cells as “iN cells” in the present study, Fig. 1D,E, p < 0.0001 and p = 0.0046, respectively). The mRNA expression level of *S100A4* (S100 Calcium Binding Protein A4: a fibroblast marker) was significantly decreased by direct conversion (Fig. 1F, p < 0.0001).

### Lower gene expression of *MEX3D* in NF1-iN cells

**Figure 2:**
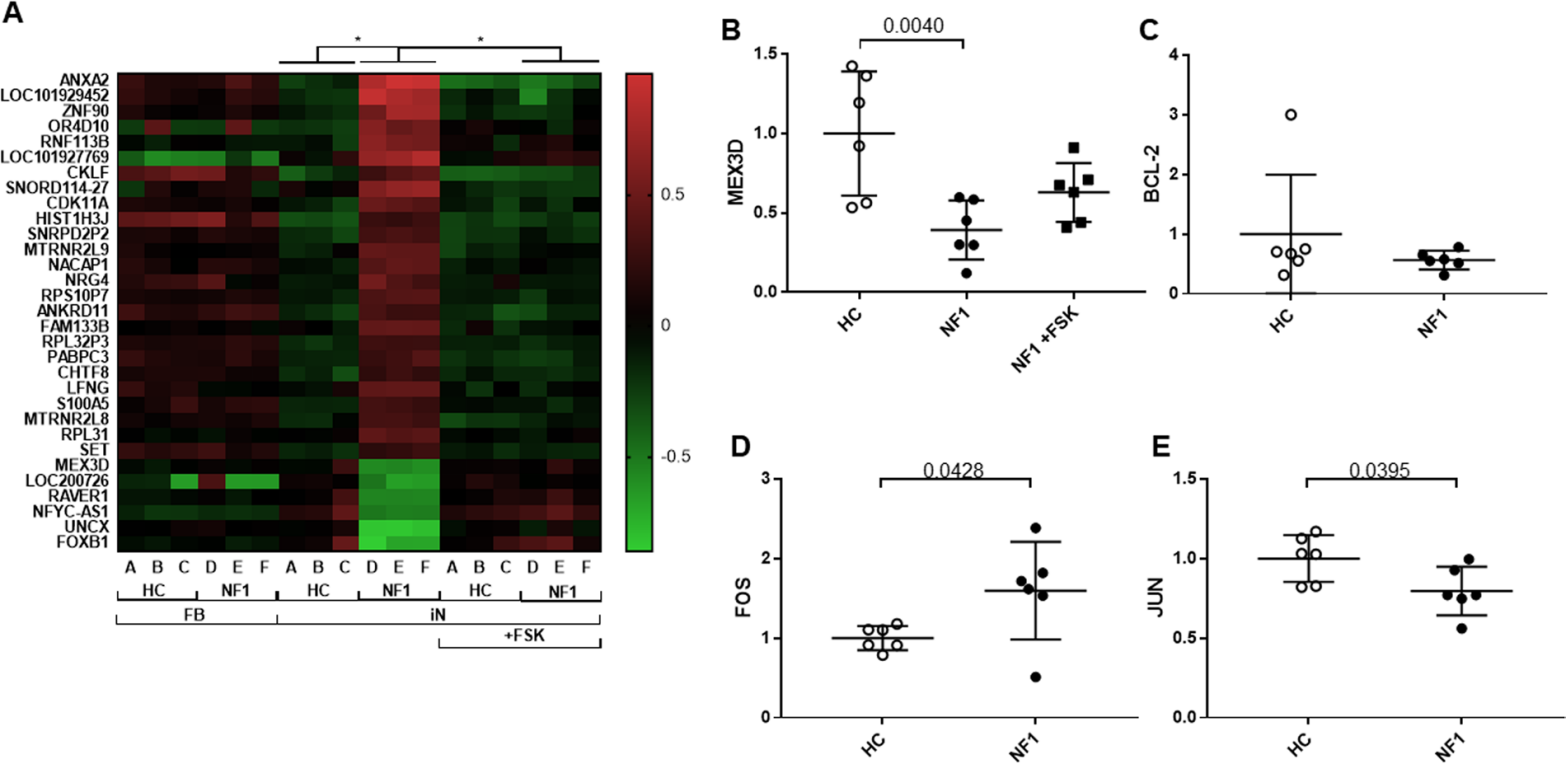
Unique gene expression profile in iN cells from patients with NF1. (**A**) Heatmap of the 31 genes that were revealed as aberrant in microarray analysis. *limma adjusted p-value < 0.05. Red indicates higher expression genes, and green indicates lower expression genes. (**B**) *MEX3D* mRNA expression level. One-way ANOVA/Tukey’s test, n = 6 each group. (**C–E**) *BCL2*, *FOS*, and *JUN* mRNA expression level, respectively. Student’s t-test, n = 6 each group. Open circles show healthy controls and filled circles and squares show NF1 patients.

The association between NF1 and ACs activity has been reported in animal model studies^14,15^, while, to our knowledge, no experimental studies exist using human neuronal cells. In order to identify how dysregulation of ACs pathway contribute to the gene expression patterns of iN cells from NF1 patients, we have added the ACs activator forskolin-applied group of the microarray analysis. At first, to explore abnormal gene expression in NF1-iN cells, we conducted an unbiased microarray analysis using SurePrint G3 Human Gene Expression Microarray 8 × 60 K v2 (Agilent Technologies). We analyzed 6 male samples including 3 HC (GM03440, KYU-165, and KYU-168) and 3 NF1 (GM00622, GM01633, and GM01859). In the fibroblasts, only *PSPHP1* (Phosphoserine Phosphatase Pseudogene 1) expression level was significantly different between HC and NF1 fibroblasts by the microarray analysis, but was not confirmed by real-time PCR (6 HC & 6 NF1; data not shown). Interestingly, in the iN cells, 149 genes were significantly different in NF1-iN cells compared to HC-iN cells (Supplementary Table 3). These results strongly suggest that abnormal gene expressions of NF1 patients are only shown in iN cells but not in fibroblasts (Supplementary Fig. 1). Significant changes in the expression level of 90 genes were observed in NF1-iN cells depending on the presence or absence of forskolin (Supplementary Table 4). Among the above149 genes (HC-iN vs NF1-iN) and 90 genes (NF1-iN vs NF-iN with forskolin), 31 genes were overlapped (Supplementary Fig. 1). Interestingly, all of their expression levels in NF1-iN cells were rescued to HC level by forskolin application (Fig. 2A). We herein focused on these 31 overlapped genes, which may be strongly dysregulated in iN cells from NF1 patients via the ACs pathway.

To confirm the validity of the above 31 gene expression differences, we reassessed the expressions of 27 genes (except 4 genes: *LOC101929452*, *LOC101927769*, *SNORD114-27*, and *MTRNR2L9*) in Fig. 2A by real-time PCR analysis with all samples (6 HC & 6 NF1) showed in Supplementary Table 1 (Fig. 2B and Supplementary Fig. 2). Interestingly, we confirmed that the *MEX3D* (Mex-3 RNA Binding Family Member D) gene expression was significantly lower in NF1-iN cells (Fig. 2B, p = 0.0040). In contrast to the microarray data only with male samples, the lower *MEX3D* expression levels in NF1-iN cells were not normalized by forskolin application in the real-time PCR analysis with male and female mix samples (Fig. 2B, p = 0.3150).

MEX3D is a member of an RNA-binding protein family with highly homologous members: MEX3 A, MEX3B, MEX3 C, and MEX3D^25^. All members of the MEX3 family contain two KH (K Homology) RNA-binding domains in the N-terminus and a RING (Really Interesting New Gene) finger domain with ubiquitin E3 ligase activity at the C-terminus. A previous study demonstrated that MEX3D enhances the degradation of *BCL2* mRNA through interacting with its AU-rich elements (AREs)^26^. Therefore, we assessed the mRNA level of *BCL2*, however there was no difference between HC- and NF1-iN cells (Fig. 2C, p = 0.3134). AREs were initially reported in the 3′-UTR (untranslated region) of the mRNAs of early response genes such as *FOS* (Fos Proto-Oncogene, AP-1 Transcription Factor Subunit), *MYC* (V-MYC Avian Myelocytomatosis Viral Oncogene Homolog), and *JUN* (Jun Proto-Oncogene, AP-1 Transcription Factor Subunit), which code for powerful transcriptional activators, and *CSF2* (Colony Stimulating Factor 2), *IL2* (Interleukin 2), *IL3*, and *IL6*, which code for growth factors and cytokines. These mRNAs are finely regulated in response to external stimuli and are subject to rapid turnover^27,28^. Thus, we next assessed whether the lower *MEX3D* in NF1-iN cells is associated with expression levels of these mRNAs. *FOS* mRNA expression level in NF1-iN cells was significantly higher compared to that in HC-iN cells (Fig. 2D, p = 0.0428). Conversely, *JUN* mRNA was significantly lower in NF1-iN cells (Fig. 2E, p = 0.0395). The expression levels of other genes (*MYC*, *CSF2*, *IL2*, *IL3*, and *IL6*) showed no significant differences (Supplementary Fig. 3). To our knowledge, there is no report that shows the direct interaction between MEX3D and *FOS* or *JUN* in neuronal cells. In order to assess whether the reduction of *MEX3D* affects the *FOS* and *JUN* mRNA expression levels in neuronal cells, we conducted *Mex3d*-knockdown experiment using a mouse neuronal cell line, Neuro2A (Supplementary Fig. 4A). Mex3d knockdown by siRNA significantly increased both *Fos* and *Jun* mRNA expression levels in Neuro2A cells (Supplementary Fig. 4B,C, p = 0.0002, 0.0360, respectively). This result suggests that a strong interaction exists between MEX3D (Mex3d) and *FOS*/*JUN* (*Fos*/*Jun*) not only in human neuronal cells but also in mouse cells, even though the direction of *JUN* (*Jun*) gene expression level was inverted. On the other hand, knockdown of *Mex3d* did not change the mRNA expression level of Nf1 in Neuro2A cells (Supplementary Fig. 4D), and knockdown of Nf1 did not change the mRNA expression level of *Mex3d* in Neuro2A cells (Supplementary Fig. 4E,F). These results suggest that the lower expression level of *MEX3D* mRNA found in NF1-iN cells is not validated in a mouse neuronal cell line, Neuro2A, suggesting the importance of analyzing human cells in disease models.

### Higher *BCL2* mRNA expression of early-stage iN cells from NF1 patients

A previous study has shown that anti-apoptotic protein BCL2 was elevated in neuronal stem cells (NSCs) from NF1-disrupted mice^12^. To our knowledge, there is no data about BCL2 aberrant in the matured neuronal cells of NF1-disrupted mice or NF1 patients. In the present study, elevation of *BCL2* mRNA was not observed in Day-14 iN cells (Fig. 2C). We thus hypothesized that upregulation of *BCL2* by NF1-disruption may be a specific developmental event in early-stage neuronal cells.

Treutlein *et al*. have shown that the initial transcriptional response of the generation of iN cells occurs relatively homogeneously among fibroblasts, but during the neuronal maturation of iN cells, a part of the induced cells population takes the alternative myogenic cell fate^29^. This should also mean that iN cells at the early-stage after transfection constitute a homogeneous population, and we further hypothesize that early-stage iN cells may show some characteristics of pre-mature neuronal cells in early developmental stage, although the conversion and neural maturation are different events.

**Figure 3:**
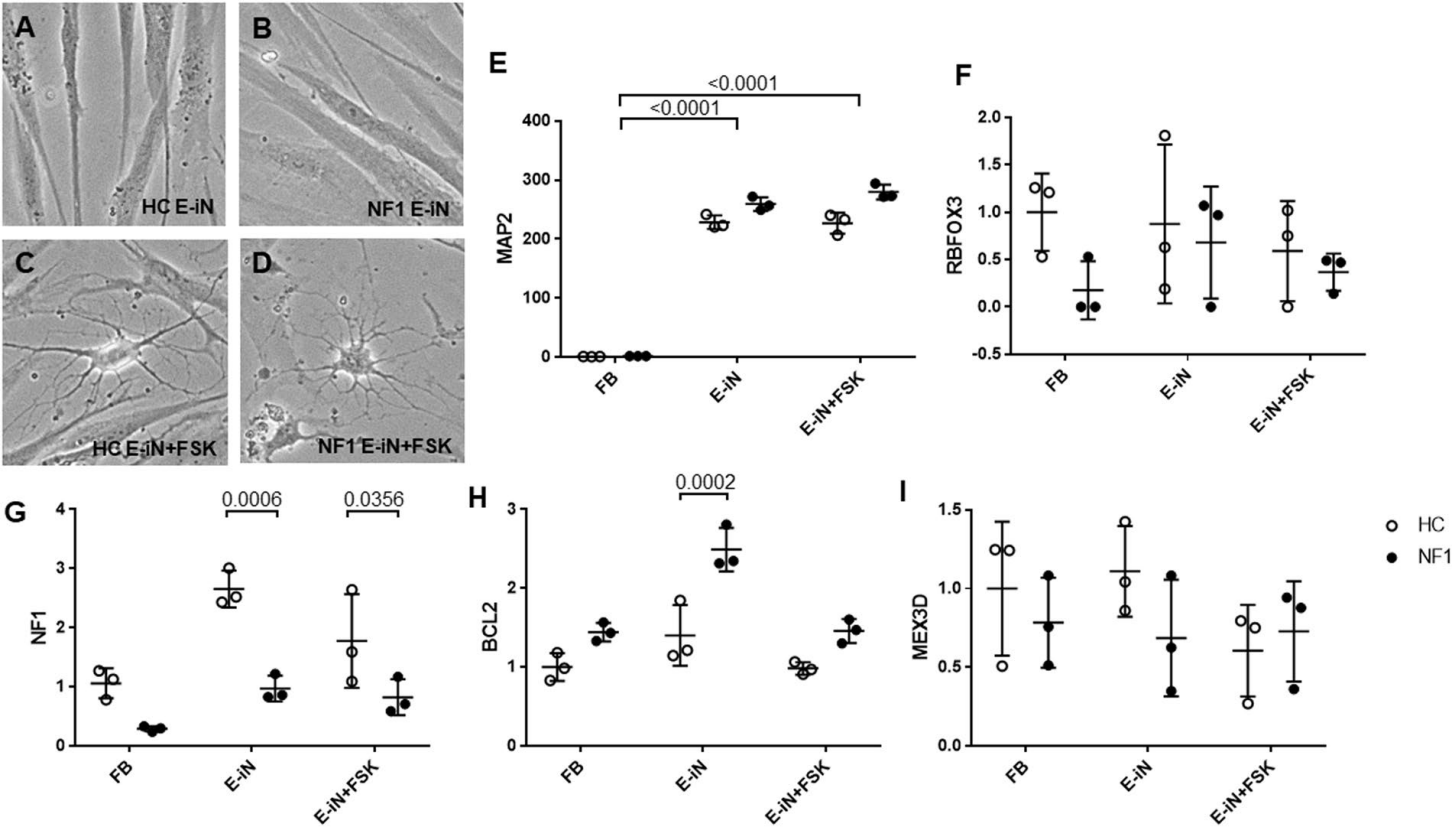
Early-stage iN (E-iN) cells at day 5 after neuronal induction. Cell morphology of Day-5 E-iN cells of HC group (**A**), NF1 group (**B**), HC group with forskolin (FSK) (**C**), and NF1 group with FSK (**D**). (**E**,**F**) *MAP2* and *RBFOX3* mRNA expression level. Two-way ANOVA/Tukey’s test, n = 3 each group. (**G–I**) *NF1*, *BCL2*, and *MEX3D* mRNA expression level. Two-way ANOVA/Sidak’s test, n = 3 each group. Open circles show healthy controls and filled circles show NF1 patients.

Morphology of Day-5 iN cells (early-stage iN cells) was not markedly different from fibroblasts (Fig. 3A,B). Surprisingly, forskolin converted iN cells from fibroblast-like shape into long-branched neuron-like morphology even at Day 5 (Fig. 3C,D). These Day-5 iN cells showed high levels of *MAP2* compared to fibroblasts, even without forskolin (Fig. 3E, p < 0.0001). As shown the above, Day-14 iN cells showed higher expression level of *RBFOX3*, a mature neuronal marker (Fig. 1E), however there were no significant differences on the expression of *RBFOX3* between fibroblasts and Day-5 iN cells with and without forskolin (Fig. 3F, p = 0.8059). Based on these data, we suppose that Day-5 iN cells may express some of the characteristics of pre-mature neuronal cells, compared to Day-14 developed-stage iN cells. *NF1* mRNA expression level of early-stage iN cells from NF1 patients was significantly lower compared to HC-iN cells (Fig. 3G, p = 0.0006). Consistent with the data from NSCs of NF1-disrupted mice, *BCL2* mRNA expression level of early-stage NF1-iN cells was significantly higher (Fig. 3H, p = 0.0002). These data partially support our hypothesis that higher functions of BCL2 is observed only on early-stage neuronal cells in NF1 patients.

## Discussion

This is the first translational study to investigate whether neuronal gene dysregulation exists in neuronal cells of NF1 patients by using human iN cells. Based on the present study, we suggest that MEX3D, a RNA-binding protein, is decreased in the neuronal cells of NF1 patients. The expression level of *Mex3d* was not affected by *Nf1* knockdown in mouse Neuro2A cells, and *vice versa*. This result may be due to the specificity of animal species or cell types. The low expression level of *MEX3D* mRNA found in NF1-iN cells may be a phenomenon specific to human neuronal cells. Further experiments using different types of animal and human cell cells should be warranted to validate our pilot findings. MEX3D is known to interact with AREs of *BCL2* mRNA, and promote the rapid decay of target mRNA at the post-transcriptional level^26^. In the present study, *BCL2* was higher only in early-stage NF1-iN cells, but not in developed-stage NF1-iN cells. Interestingly, a previous study showed the increased BCL2 in NSCs from the NF1-disrupted mice^12^. mRNA and protein of BCL2 exist at relatively high levels during the nervous system development, and decrease in the postnatal brain^30–32^. Abnormalities of apoptosis constitutes a pathogenesis of neurodevelopmental disorders^33^. The majority of neuronal cells are immature or premature in neurodevelopmental stages, and apoptosis of immature/premature neuronal cells must be highly regulated to form appropriate neural circuitry. Therefore, there is a possibility that BCL2-mediated neuronal apoptosis may be disturbed and this may form abnormal neural circuitry in the brain of NF1 patients during neurodevelopmental periods. This disturbed pathway may be one of the pathogenic mechanisms of ASD and other neurodevelopmental disorders in NF1 patients.

A previous study demonstrated that MEX3D interacts with AREs^26^. AREs were initially reported in the 3′-UTR of the mRNAs of early response genes such as *FOS*, *MYC*, and *JUN*, which code for powerful transcriptional activators, and *CSF2*, *IL2*, *IL3*, and *IL6*, which code for growth factors and cytokines. These mRNAs are finely regulated in response to external stimuli and are subject to rapid turnover^27,28^. We observed the higher level of *FOS* mRNA in NF1-iN cells (Fig. 2D). Higher expression level of *FOS* in human NF1-iN cells was also supported using *Mex3d* knockdown cells in mouse Neuro2A cells. This is considered to be a more general and direct phenomenon regardless of animal species or cell types. There is a possibility that MEX3D suppresses the *FOS* level by interacting with AREs. A previous study demonstrated that an animal model of NF1, NPcis mice, showed high levels of FOS expression in their tumors and brain^34^. Both the cAMP-CREB and RAS-MAPK pathways can stimulate the expression of FOS^35^. Here, we propose a novel mechanism of FOS enhancement in NF1 neuronal cells, in which MEX3D depletion may elongate the half-life period of *FOS*. In addition, FOS is known to have an anti-apoptotic role especially in neuronal cells^36,37^. We observed the decrease of *JUN* mRNA expression in the NF1-iN cells. *Mex3d* knockdown by siRNA significantly increased *Jun* mRNA expression level in Neuro2A cells. This data did not match with the result in NF1-iN cells. However, this result is reasonable considering the function of Mex3d and ARE of *Jun* mRNA. In NF1-iN cells, some stronger factors other than MEX3D seem to suppress the expression level of *JUN* mRNA. JUN has a pro-apoptotic function in neuronal cells^38^. In sum, we propose that both FOS and JUN act to suppress the apoptosis of neuronal cells synergically in NF1 patients. In order to confirm the validity of the above results, further verification is necessary using *NF1* and/or *MEX3D* knockdown iN cells or iPS cell-derived neurons. Mechanism studies regarding the association between NF1 and related genes and additional investigations for functional study using multiple methods (such as apoptosis analysis and protein level analysis) are needed to validate the present pilot data and to clarify our proposed hypothesis.

Recent epidemiological studies have shown that NF1 patients are highly comorbid with ASD, and prevalence of ASD is about two times higher in males than females^9,39^. In the present study, microarray analysis with 3 HC & 3 NF1 male samples showed that 149 gene expression levels was different in male NF1-iN cells compared to male HC-iN cells, and 31 gene expression levels of them were rescued by forskolin application. Furthermore, all of their expression levels in NF1-iN cells were rescued to HC level by forskolin application (Fig. 2A). However, it is important to note that many differences were not replicable when we validated these results with a 6 HC & 6 NF1 real-time PCR analysis that included female samples (Supplementary Fig. 2). Further investigation with more samples will clarify our novel hypothesis that neuronal pathologies tend to be expressed especially in male NF1 patients, which may help to understand the sex-specific differences in ASD and other neuropsychiatric disorders.

## Conclusion

Using the human iN technique, we are the first to report lower expression of *MEX3D* in iN cells from NF1 patients. Furthermore, we revealed that the expression of *FOS* and *BCL2* mRNA, which act as anti-apoptotic in neuronal cells, were higher in developed- and early-stage iN cells of NF1 patients, respectively. Thus, apoptosis of neuronal cells during neurodevelopmental period may be disturbed in NF1 patients. The present study has suggested that analysis of early-stage iN cells may reflect characteristics of premature neuronal cells during neurodevelopmental periods, and we thus believe that molecular analysis of not only developed-stage but also early-stage iN cells may explore the novel pathophysiology of neuronal cells in various neurodevelopmental disorders including ASD and schizophrenia. However, meanings of early-stage iN analysis have not been well clarified. Thus, further translational studies using induced pluripotent stem (iPS) cell-derived neuronal stem cells and neuronal cells of step-by-step developmental-stages are needed to confirm meanings of analysis using early-period iN cells and to validate our preliminary findings. Moreover, the present findings based on gene expression analysis should be validated by additional analysis such as apoptosis analysis, protein level analysis and neuron functional assays. Since we used mixed cultures, some neuronal down-regulations of mRNA may be masked by contamination of fibroblasts. Thus, further experiments are warranted using much more purified iN cells from mix cultures and/or a single cell analysis. Especially, gene-expression analysis of single cell level (single cell PCR) are warranted to validate our findings. On the other hand, deeper molecular mechanisms, especially the interaction between NF1, MEX3D, FOS, JUN, and BCL2, should be addressed. Our additional *in vitro* study using mouse Neuro2A cells did not show some interactions found by the gene expression analysis of human NF1-iN cells, for example between *Nf1* and *Mex3d*, thus these interactions might be human specific, indicating the importance of human cellular model studies. Human iPS cell-derived neuron studies are expected to confirm the present findings. In addition, further cellular analysis especially considering sex-specific neuronal dysregulation should be conducted to reveal unknown neurobiological roles of sex underlying the pathophysiology of ASD.

On the other hand, nerve sheath tumor and/or glioma, which are the main diseases of NF1, are known to arise from glial cells but not from neuronal cells. Thus, further investigations using human derived glial cells (*e*.*g*. human iPS cell-derived glial cells) are also needed to clarify underlying pathophysiology of NF1 from the perspective of glia.

Finally, we propose that administration of the drug forskolin and/or other AC activators, which can be easily delivered into the brain, to NF1 patients (especially males) during the early developmental period should be further studied towards preventing the occurrences of ASD and neuropsychiatric disorders in later life.

## Electronic supplementary material

Supplemental information

Supplementary Table 3

Supplementary Table 4
