# Supplementary material for: Dysregulated gene expressions of MEX3D, FOS and BCL2 in human induced-neuronal (iN) cells from NF1 patients: a pilot study
Source: Sci Rep. 2017 Oct 24;7:13905. doi: 10.1038/s41598-017-14440-7 (PMC5654769; doi:10.1038/s41598-017-14440-7)
Supplement: Supplementary file 1 — Supplemental information [file 41598_2017_14440_MOESM1_ESM.pdf]

2017.09.13. Revise

Journal: Scientific Reports

Title (19 / 20 words)

Dysregulated gene expressions of *MEX3D*, *FOS* and *BCL2* in human induced-neuronal (iN) cells from NF1 patients: a pilot study

Authors: Noriaki Sagata [1], Takahiro A. Kato [1#], Shin-ichi Kano [2], Masahiro Ohgidani [1], Norihiro Shimokawa [1], Mina Sato-Kasai [1], Kohei Hayakawa [1], Nobuki Kuwano [1], Ashley M. Wilson [2], Koko Ishizuka [2], Shiori Kato [3], Takeshi Nakahara [3], Makiko Nakahara-Kido [3], Daiki Setoyama [4], Yasunari Sakai [5], Shouichi Ohga [5], Masutaka Furue [3], Akira Sawa [2], Shigenobu Kanba [1]

Affiliations:

1) Department of Neuropsychiatry, Graduate School of Medical Sciences, Kyushu University, Maidashi 3-1-1, Higashi-ku, Fukuoka 812-8582, Japan

2) Departments of Psychiatry, Mental Health, Neuroscience, and Biomedical Engineering, Johns Hopkins University School of Medicine and Bloomberg School of Public Health, 600 North Wolfe St., Baltimore, MD 21287, USA

3) Department of Dermatology, Graduate School of Medical Sciences, Kyushu University, Maidashi 3-1-1, Higashi-ku, Fukuoka 812-8582, Japan

4) Department of Clinical Chemistry and Laboratory Medicine, Graduate School of Medical Sciences, Kyushu University, Maidashi 3-1-1, Higashi-ku, Fukuoka 812-8582, Japan

5) Department of Pediatrics, Graduate School of Medical Sciences, Kyushu University, Maidashi 3-1-1, Higashi-ku, Fukuoka 812-8582, Japan

#Correspondence to: Takahiro A. Kato, M.D., Ph.D. (Associate Professor; takahiro@npsych.med.kyushu-u.ac.jp)

Contents:

Supplemental information: 4 Figures and 4 Tables (These are included in the following files: Supplemental information (Sagata et al.).pdf, NF1 iN – Supplementary Table 3.xlsx, and NF1 iN – Supplementary Table 4.xlsx.)

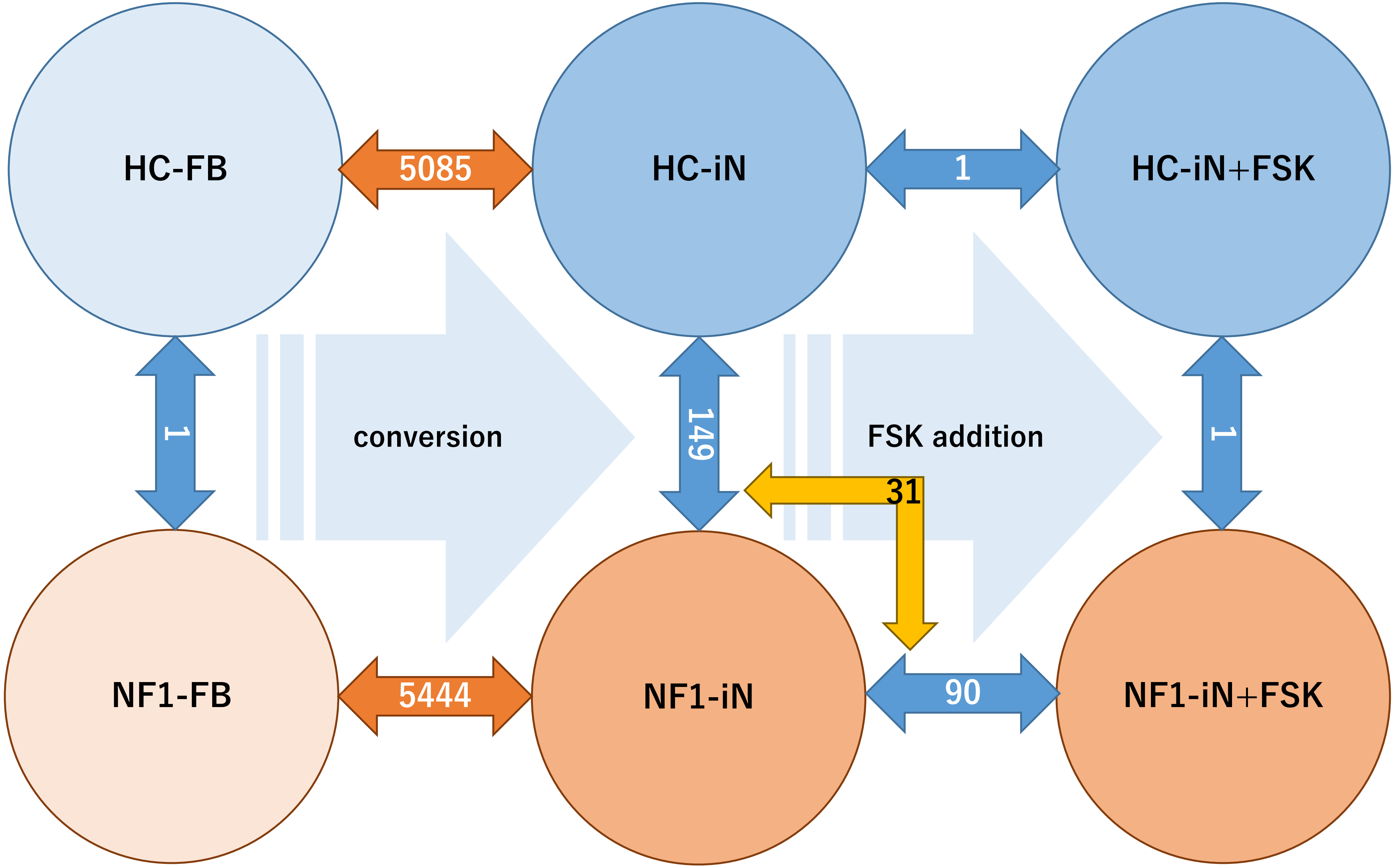

Supplementary Figure 1

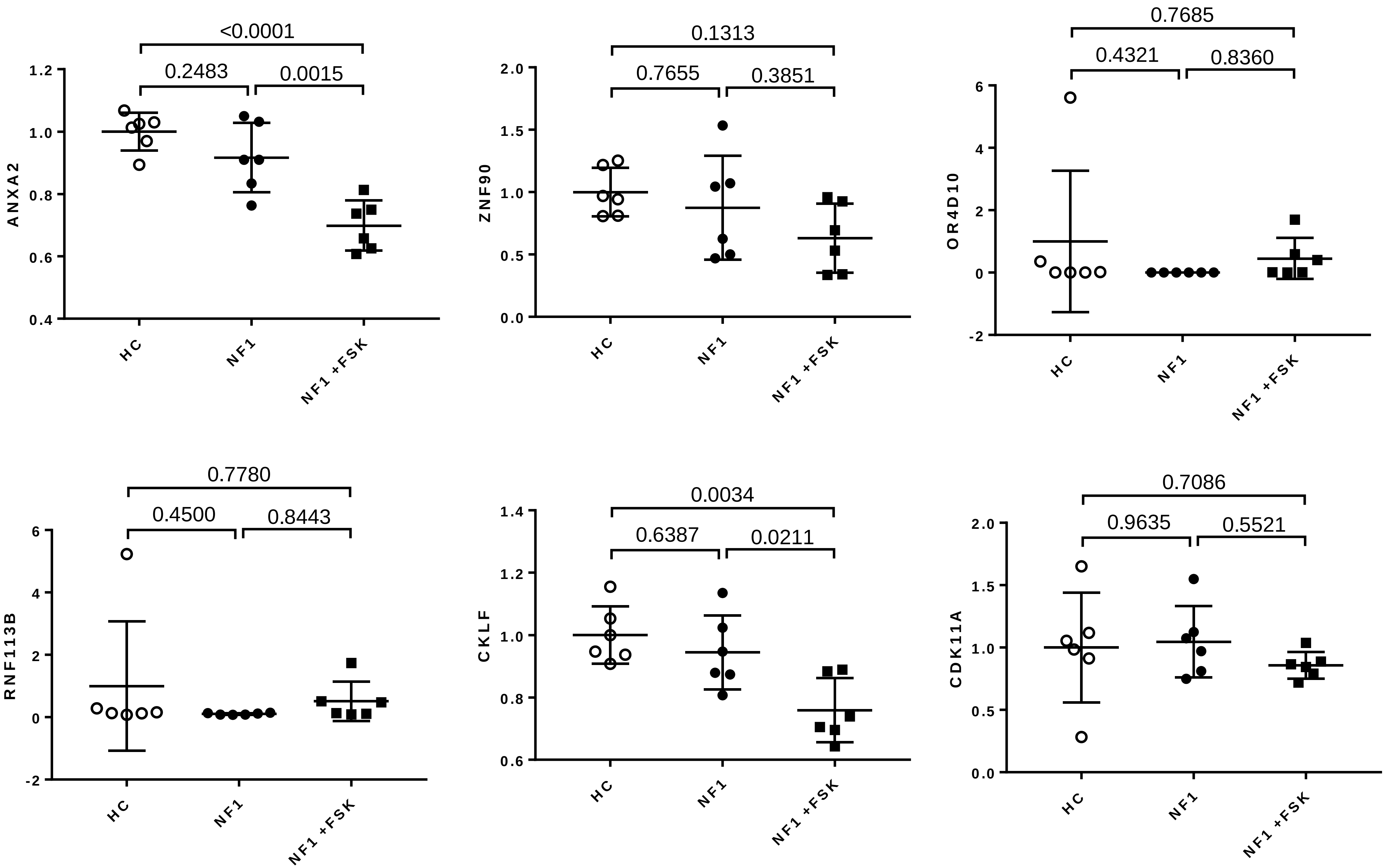

Supplementary Figure 2

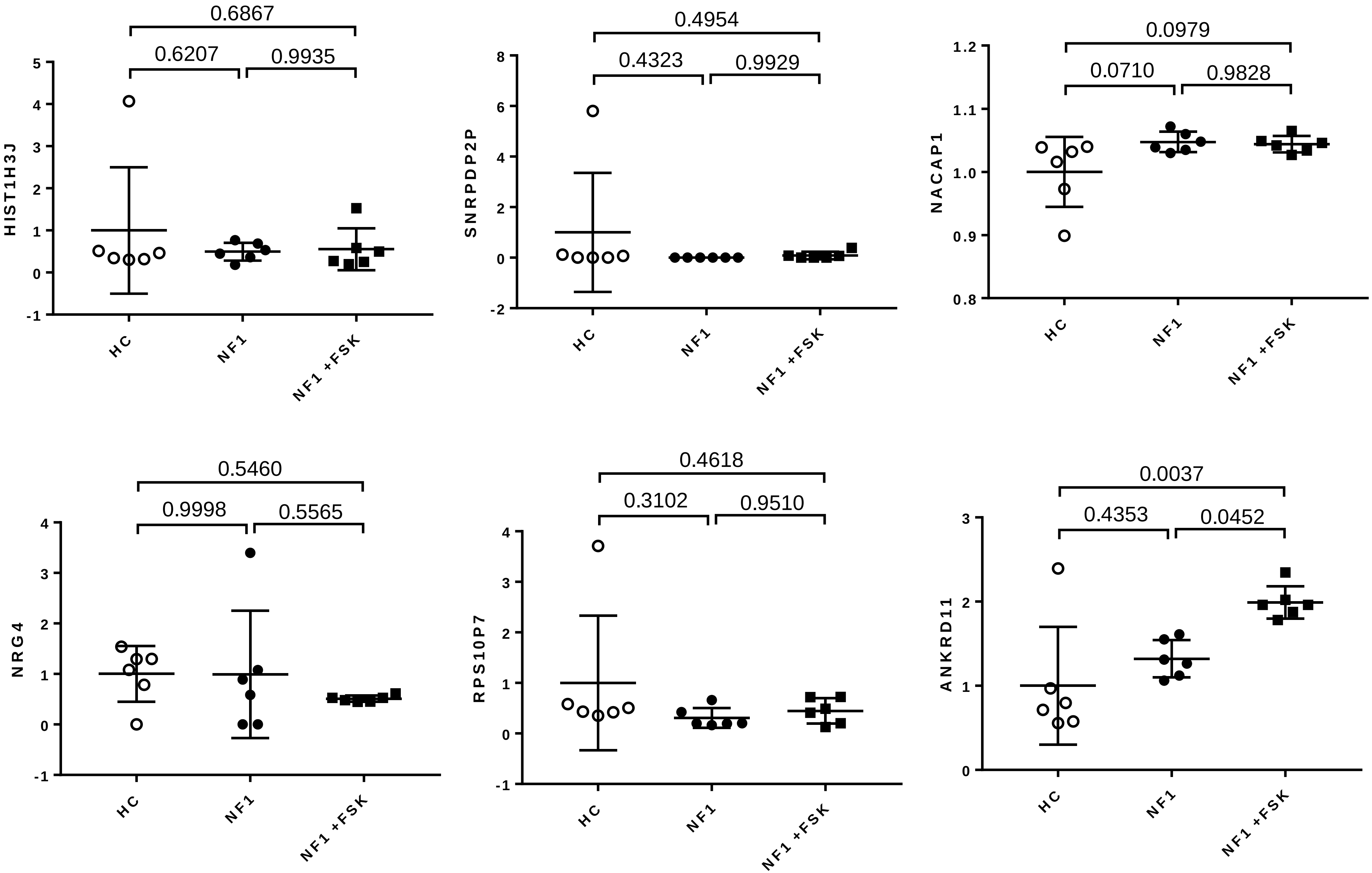

Supplementary Figure 2 (continued)

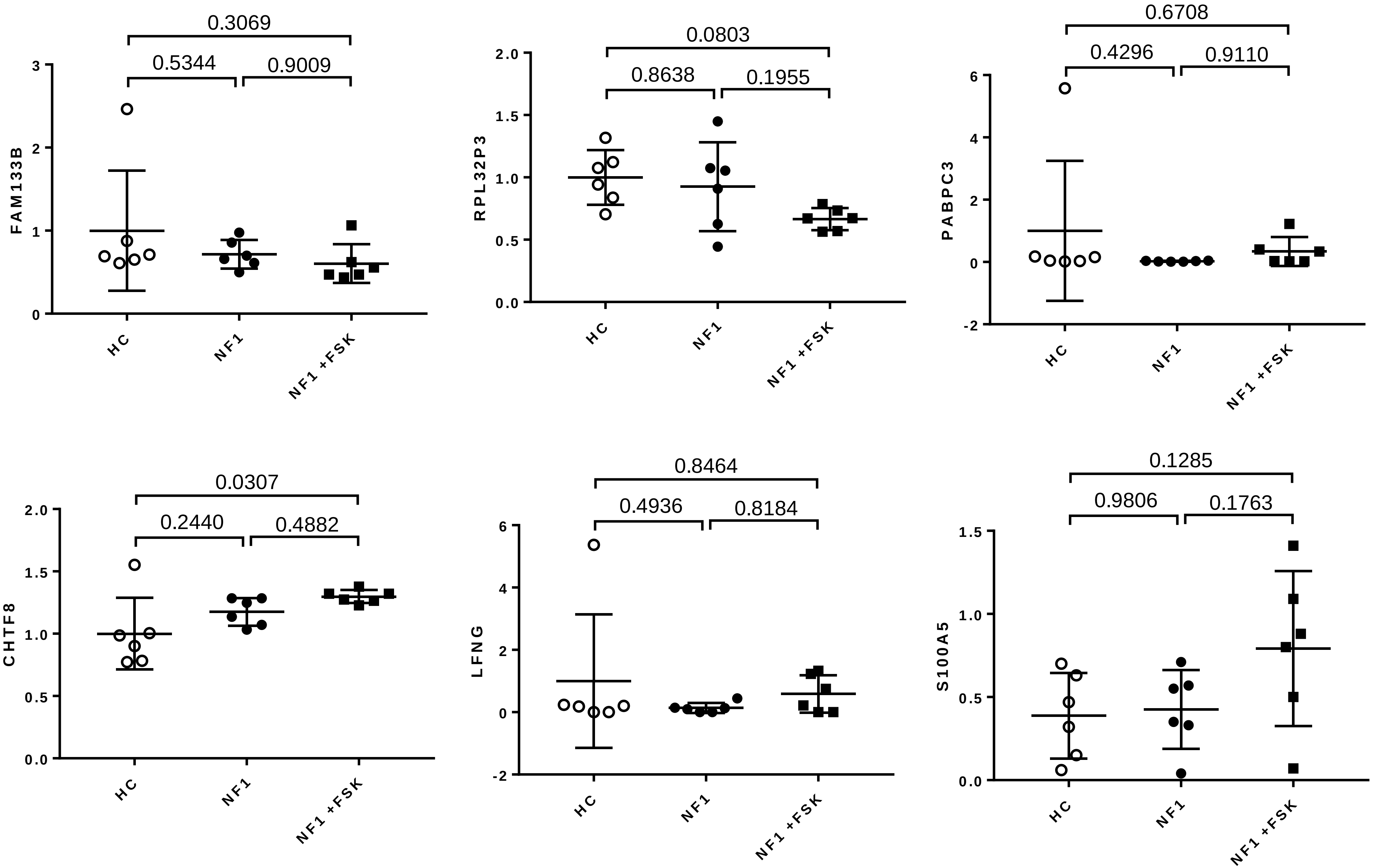

Supplementary Figure 2 (continued)

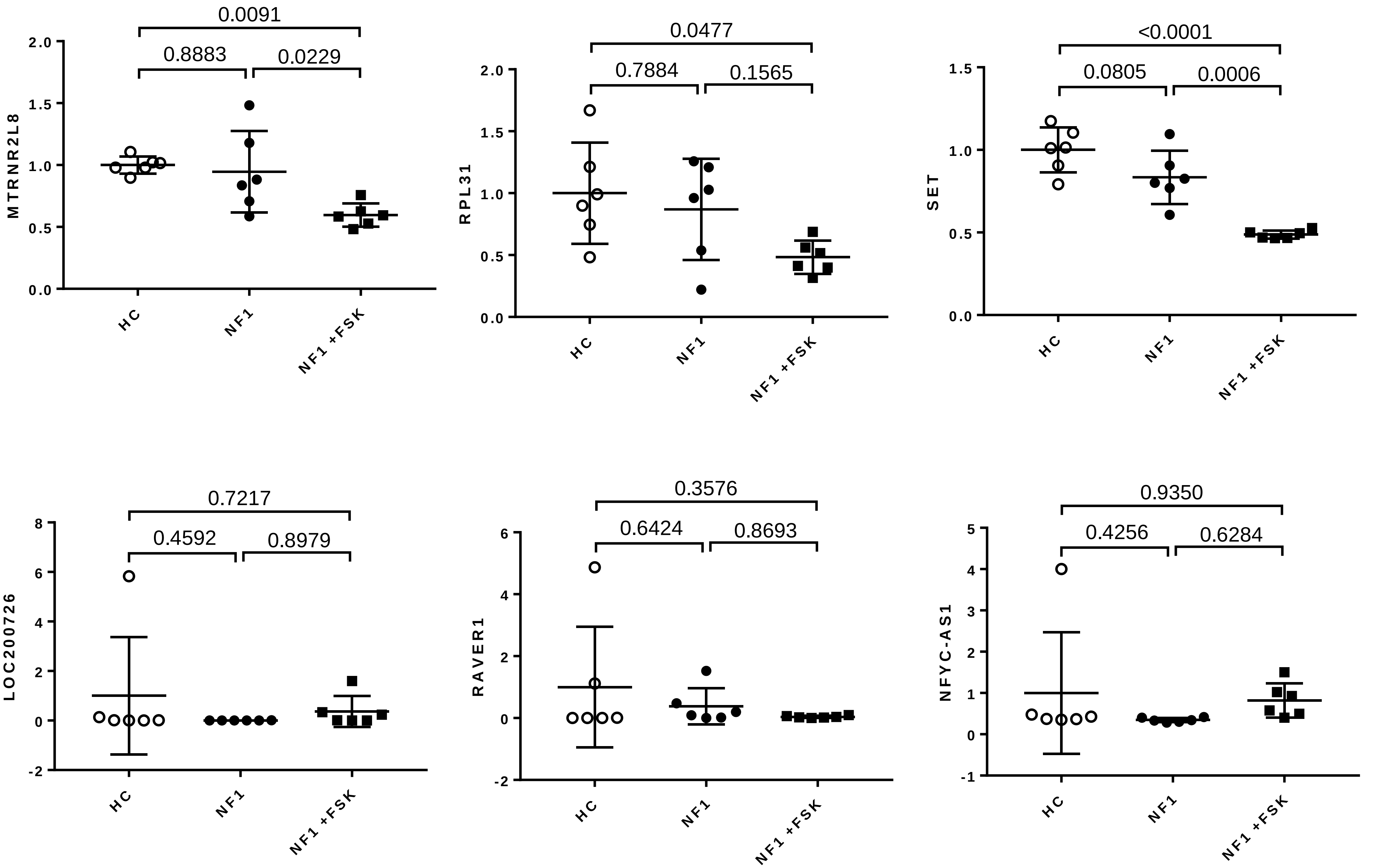

Supplementary Figure 2 (continued)

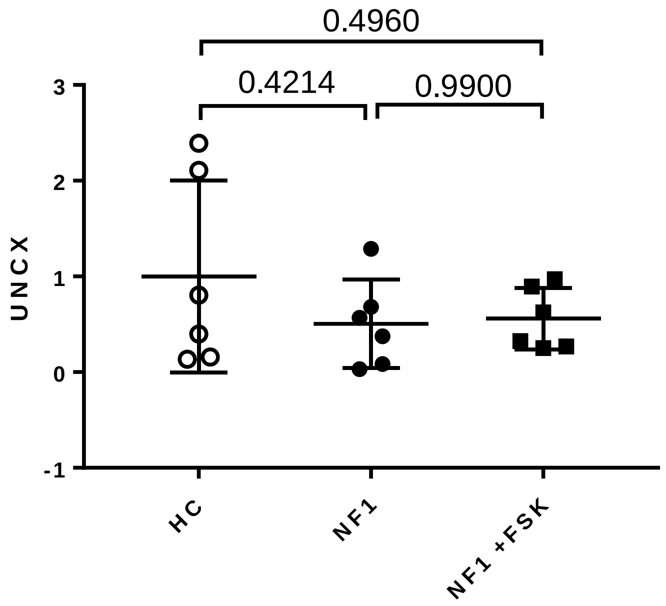

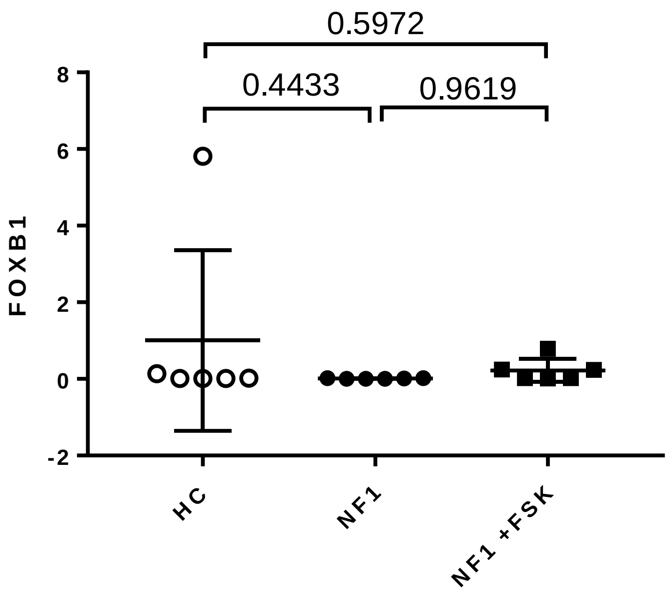

Supplementary Figure 2 (continued)

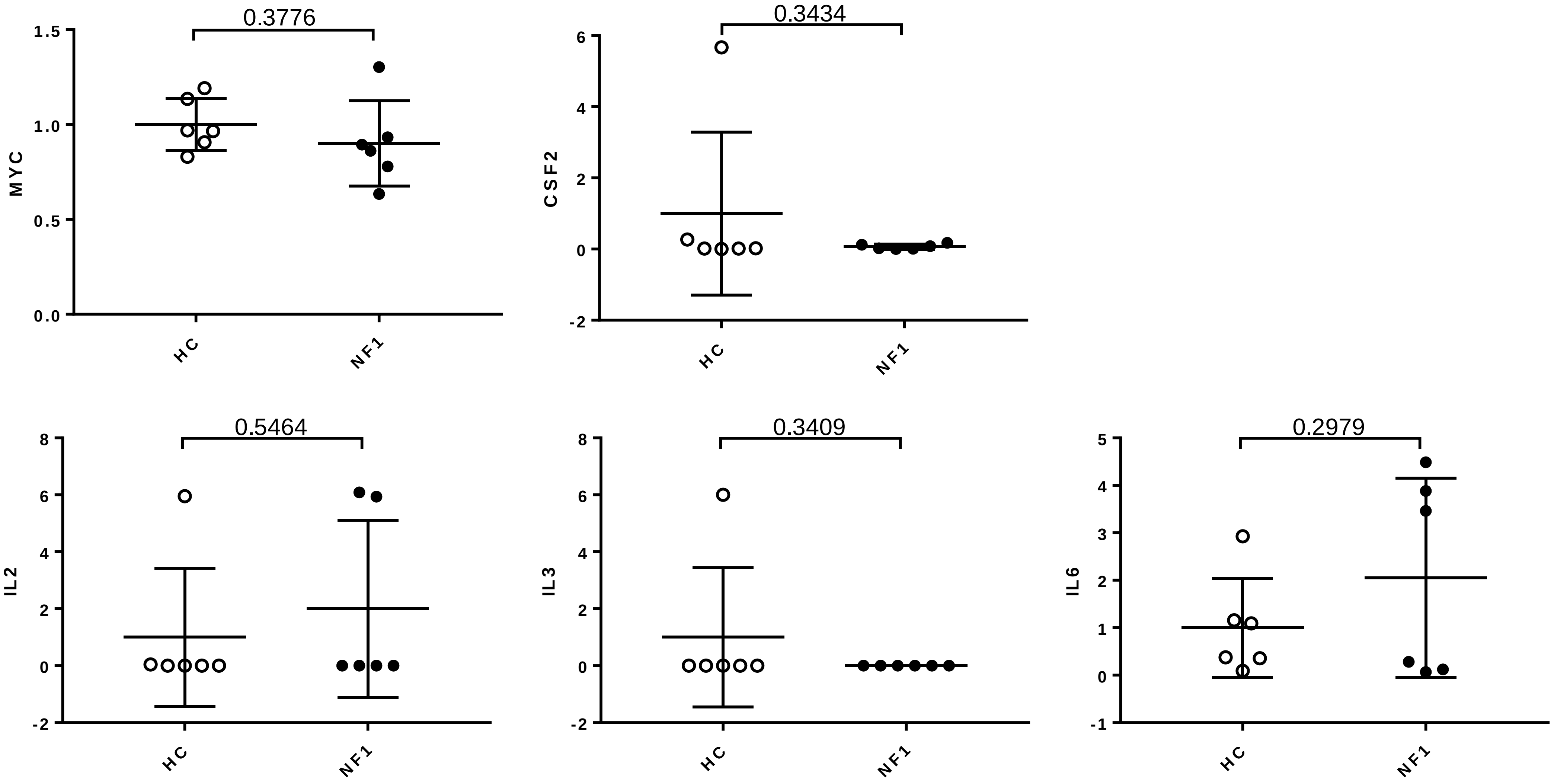

Supplementary Figure 3

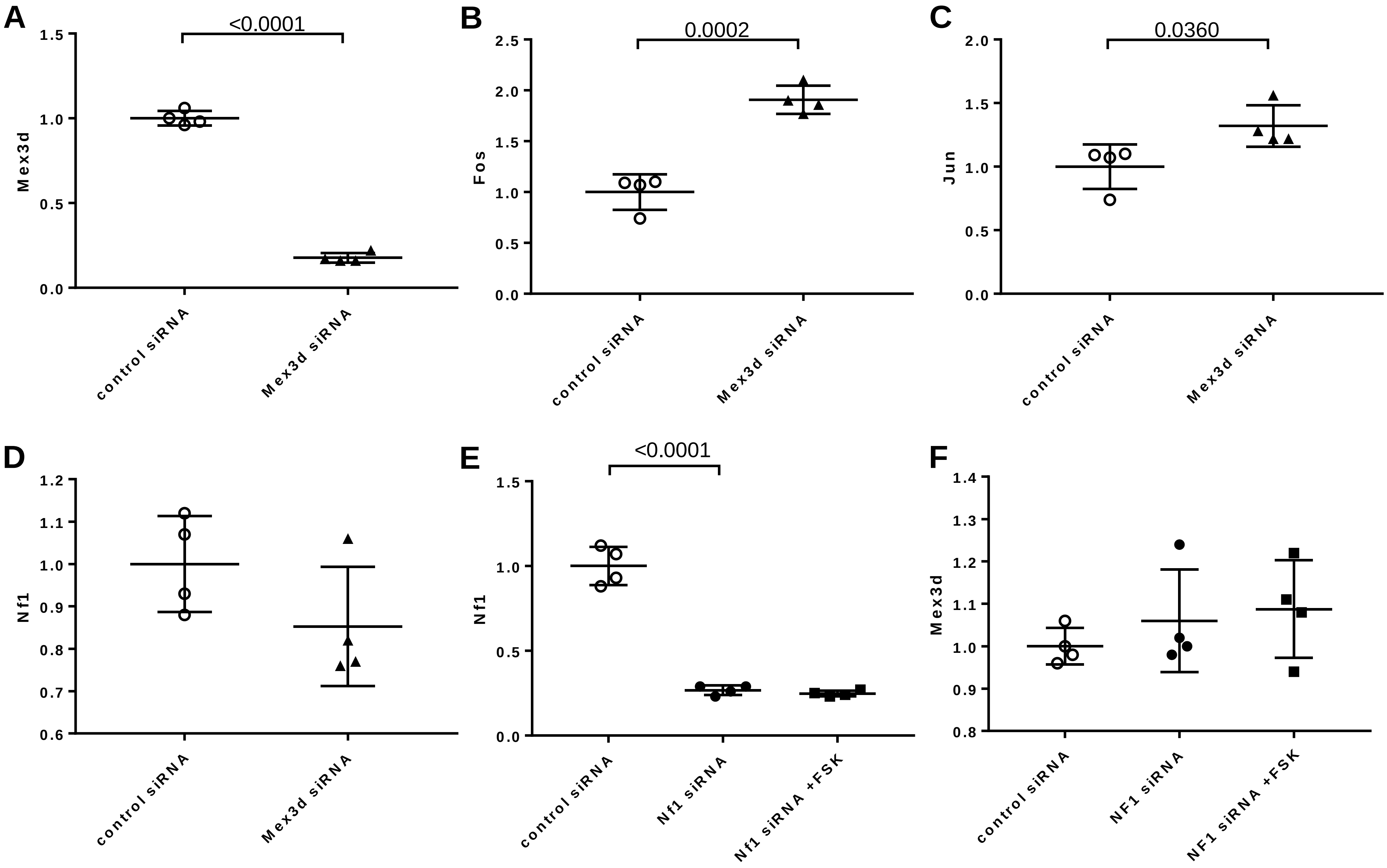

Supplementary Figure 4

Supplementary Table 1: Information of fibroblasts for generating iN cells.

Description	ID	Sex	Age
Healthy control	GM03440*	Male	20
	GM05399	Male	1
	KYU-165*	Male	21
	KYU-166	Male	22
	KYU-168*	Male	61
	KYU-176	Male	49
NF1	GM00622*	Male	8
	GM01633*	Male	61
	GM01639	Female	19
	GM01859*	Male	41
	KYU-101	Female	67
	KYU-177	Female	35

(*Samples for microarray analysis)

Supplementary Table 2: Information of the primer and probe number for quantitative real-time PCR.

Species	Target	Position	Tm	%GC	Sequence	Probe#
human	ANKRD11	5202 - 5220	60	58	accagcttcgagaggatgc	85
		5255 - 5272	60	61	ctctccttgtgccgcttg	
human	ANXA2	859 - 879	59	48	ggaaagcatcaggaaagaggt	85
		913 - 934	59	45	cttgttctgaatgcactgaacc	
human	BCL2	2335 - 2354	59	60	agagcagtagaggggtgtgg	57
		2380 - 2397	59	56	tgaaaacgggcctacctg	
human	CDK11A	2462 - 2479	59	61	accacaggcgtggctact	19
		2512 - 2533	60	45	catggagcacaaagtaagacga	
human	CHTF8	1448 - 1468	60	57	caaactcgggtcctagctctc	81
		1497 - 1515	59	53	agcccttgacatgggagat	
human	CKLF	442 - 462	59	48	ttggcactgataccagaaacc	22
		487 - 507	60	48	tgctgtcacaagtgcaaacac	
human	CSF2	326 - 345	59	55	cagccactacaagcagcact	3
		456 - 473	59	67	ccggtctcactcctggac	
human	FAM133B	1139 - 1162	59	33	tttttctcttccagtgcttgataa	14
		1185 - 1211	59	41	cccaaatatcttaaccactagtacgac	
human	FOS	292 - 311	60	60	ctaccactcacccgcagact	67
		345 - 363	59	58	aggtccgtgcagaagtcct	
human	FOXB1	784 - 801	60	56	atccccgtgcccattaag	27
		852 - 870	59	58	cgagttcgagagcaaggtg	
human	HIST1H3J	342 - 361	59	50	cgccaagcgtgtcactatta	42
		404 - 425	59	50	gatagagcaggggattatgctc	
human	IL2	261 - 281	59	48	aggccacagaactgaaacatc	65
		332 - 354	59	35	aagtgaaagtttttgctttgagc	
human	IL3	164 - 190	59	30	catgatcgatgaaattataacacactt	83
		271 - 288	60	61	cctccaggtttggccttc	
human	IL6	109 - 128	59	55	caggagcccagctatgaact	7
		182 - 199	60	61	gaaggcagcaggcaacac	
human	JUN	3012 - 3032	59	43	ccaaaggatagtgcgatgttt	19
		3054 - 3073	60	60	ctgtccctctccactgcaac	
human	LFNG	1280 - 1298	60	63	cctggagagcaggagcagt	23
		1333 - 1351	59	58	caagcgtctggatctctgc	
human	LOC200726	4-21	60	61	aagctggggacagccact	55
		56 - 76	59	52	tggagagagttccctcgagta	
human	MAP2	188 - 214	59	33	cgaactttatattttaccacttccttg	2
		244 - 263	60	50	ccgttcatctgccattcttc	
human	MEX3D	663 - 680	60	61	gccggtcttcatcgtgac	12
		707 - 725	59	58	gacaggatctcacgcttgg	
human	MTRNR2L8	423 - 444	59	50	gaggaacagctctttggacact	83
		513 - 532	60	45	tcgagcttgaacgctttctt	
human	MYC	514 - 533	59	50	gctgcttagacgctggattt	66
		569 - 586	60	56	taacgttgaggggcatcg	
human	NACAP1	344 - 363	60	55	tgtctacaagagcccgcttc	60
		409 - 429	60	52	gctgctagttgtgcttcctga	
human	NF1	2314 - 2338	60	40	ccatggatcatgaagaattactacg	1
		2385 - 2404	59	55	ctgcatcctgctgcactatc	
human	NFYC-AS1	300 - 319	59	55	aagtgaggtctctggcgaag	70
		355 - 373	59	58	tctgtgcgagggtgatagg	
human	NRG4	242 - 263	59	45	ggggctttgttatgtgatacct	23
		284 - 307	59	42	cctgtatagttttcaacgcaccta	
human	OR4D10	418 - 435	60	50	cattgcattgggctcaca	27
		463 - 482	59	50	gaaatctgcacgatggagtg	
human	PABPC3	1407 - 1425	59	63	gcccaggtgctcctagagt	54
		1456 - 1476	60	52	gacatgactcgtggaacctgt	
human	PSPHP1	347 - 365	60	58	ctcctgggttcaggcagtt	10
		406 - 425	60	45	tcctccaaagccaatgaaag	
human	RAVER1	2816 - 2834	59	58	ccccagctctgattccttc	49
		2865 - 2883	60	47	tgtttcaaaccgcaaggtg	
human	RBFOX3	751 - 768	60	61	ggggaacccctacaccaa	11
		798 - 817	59	55	gaattcaggcccgtagactg	
human	RNF113B	1035 - 1056	59	50	gcagcctgttgagtaagatgag	4
		1081 - 1102	59	45	ttggttacatactcaggcatgg	
human	RPL31	4-23	60	55	cgacccggaagttgtacttg	7
		98 - 115	59	56	tctcgccacccttctttg	
human	RPL32P3	43 - 64	59	45	ccagtcagaccgatatgtcaaa	31
		134 - 153	60	45	tgctgggcatcatatcaaga	
human	RPS10P7	104 - 123	59	50	gaaagaacgtgcctttccag	83
		154 - 176	60	43	ccagttcttcttaggcatcaaca	
human	S100A4	138 - 158	60	48	gggtgacaagttcaagctcaa	17
		211 - 232	59	41	aagcagcttcatctgtcctttt	
human	S100A5	180 - 200	59	48	agggacagagatcagggaaat	8
		278 - 298	59	57	ccagaggagtctccatcacag	
human	SAMD3	925 - 942	59	56	cgtggttaatgccctgct	76
		982 - 1002	59	43	agggctcgtttccataaaaag	
human	SET	2367 - 2387	59	38	aaaattggccttttacctgga	87
		2415 - 2434	60	55	gagcaccaggtctgttggat	
human	SNRPD2P2	532 - 551	60	60	gcagggactcagtcatcacc	10
		605 - 625	59	52	ccccataacaaagggtaggtc	
human	UNCX	1850 - 1875	59	35	aagagtaaacgaaagtgctgtatgaa	27
		1896 - 1914	59	63	gtctcacccccagtcctgt	
human	ZNF90	349 - 366	59	50	gattgccaaatccccaga	19
		399 - 417	59	53	acacagagattgcgctgct	
mouse	Fos	210 - 230	59	57	gggacagcctttcctactacc	67
		278 - 297	60	50	agatctgcgcaaaagtcctg	
mouse	Jun	154 - 173	59	45	tttctcaccaactgcttgga	92
		199 - 218	59	45	ccaaatgctccccaaaatac	
mouse	Mex3d	1562 - 1579	60	61	gccttcgaggggataacg	71
		1607 - 1624	59	61	ggctgcccacatagaagg	
mouse	Nf1	10872 - 10892	59	48	tcccccaaaggagactctaat	68
		10920 - 10941	59	36	ccaaattgtgtaaaatgggaca	

Supplementary Figure 1: Simplified schematic of microarray analysis. Circles show 6 sample groups; healthy control fibroblast (HC-FB), NF1 patient fibroblast (NF1-FB), healthy control iN cells (HC-iN), NF1 patient iN cells (NF1-iN), healthy control iN cells with forskolin (HC-iN+FSK), and NF1 patient iN cells with forskolin (NF1-iN+FSK). Blue and orange double arrows indicate the number of aberrant genes between two groups (circles). A yellow double arrow indicates the number of overlapping genes between two blue double arrows.

Supplementary Figure 2: Quantification of mRNA expressions by real-time PCR. There were no significant changes on the expressions of 26 genes of Day-14 iN cells between HC and NF1 group. One-way ANOVA / Tukey's test, n = 6 each group. Open circles show healthy controls and filled circles and squares show NF1 patients.

Supplementary Figure 3: Quantification of AREs-including mRNA expressions by real-time PCR. There were no significant differences on the expressions of 5 genes of Day-14 iN cells between HC and NF1 group. Student's t-test, n = 6 each group. Open circles show healthy controls and filled circles show NF1 patients.

Supplementary Figure 4: Quantification of mRNA expressions of Neuro2A cells by real-time PCR. (**A-D**) *Mex3d*, *Fos*, *Jun*, and *Nf1* mRNA expression level, respectively. Student's t-test, n = 4 each group. (**E-F**) *Nf1* and *Mex3d* mRNA expression level, respectively. One-way ANOVA / Tukey's test, n = 4 each group. Open circles show control siRNA, filled triangles show *Mex3d* knockdown using siRNA, and filled circles and squares show *Nf1* knockdown using siRNA.

Supplementary Table 1: Supplementary Table 1. Information of fibroblasts for generating iN cells.

Supplementary Table 2: Information of the primer and probe number for quantitative real-time PCR.

Supplelmentary Table 3: Significantly different 149 gene expressions in NF1-iN cells compared to HC-iN cells determined by microarray analysis.

Supplelmentary Table 4: Significantly different 90 gene expressions in NF1-iN cells compared to NF1-iN cells with forskolin determined by microarray analysis.
